# A generalized electrode mechanism unifying classical electrochemical pathways in square-wave voltammetry

**DOI:** 10.1038/s41598-026-60887-y

**Published:** 2026-07-07

**Authors:** Rubin Gulaboski, Ivan Bogeski

**Affiliations:** 1 Faculty of Medical Sciences, Goce Delcev University, Stip, Macedonia; 2 Molecular Physiology, Institute of Cardiovascular Physiology, University Medical Center Göttingen, Humboldtallee 23, 37073 Göttingen, Germany

**Keywords:** Multistep electrode mechanisms, General electrochemical framework, EC, CE and EC’ mechanisms, Voltammetric simulations, Square-wave voltammetry, Biophysics, Chemistry

## Abstract

The electrochemical framework consisting of two successive electron-transfer steps (EE) separated by a reversible homogeneous chemical equilibrium (Crev), followed by an irreversible regenerative chemical reaction (C′) linked to the participants in the second electron-transfer step is assigned as ECrevEC′ mechanism. The ECrevEC′ electrochemical scheme can be considered as a comprehensive and unifying mechanism that covers a broad spectrum of important classical electrochemical mechanisms as limiting cases. Depending on the magnitude of equilibrium constant of the intermediate chemical step (*K*eq), the associated chemical rate parameter governing this equilibrium (*k*_chem_), and the kinetic rate constant of the irreversible regenerative reaction (*k*_c_), the ECrevEC′ mechanism can coherently reproduce the features of the simple “E mechanism”, as well as the “ECrev”, “CErev”, “EC′ (regenerative)”, and also the “EE”, “ECrevE” and EEC’ mechanisms. Theoretical treatment of this complex reaction scheme is of fundamental importance, as it enables a systematic and unified exploration of the entire mechanistic landscape within a single mathematical formalism. Mathematical modeling of this complex mechanism affords detailed insight into the coupled effects of electron-transfer kinetics, chemical equilibria, and homogeneous regeneration reactions. Voltammetric simulations based on the ECrevEC′ mechanism exhibit rich and valuable diagnostic features in both forward and backward current responses, permitting important mechanistic distinctions to be resolved via analysis of relevant parameters of the voltammetric patterns. The ECrevEC’ framework provides a powerful tool for the interpretation of experimental data in complex voltammetric systems involving multiple coupled chemical reactions. This electrochemical scheme is widely applicable theoretical platform with high relevance to redox catalysis, bioelectrochemistry and energy-conversion systems.

**Supplementary Information:**

The online version contains supplementary material available at 10.1038/s41598-026-60887-y.

## Introduction

By knowing the fact that the majority of electron-transfer processes are rarely elementary^1–9^, mathematical modeling of multistep electrode mechanisms under voltammetric conditions plays crucial role in today’s electrochemistry and bioelectrochemistry. In most of the experimental scenarios, electrochemical processes are coupled with chemical transformations occurring either before, after, or between two consecutive electron transfer steps^8–15^. In biochemical and physiological systems^9,14–17^, for example, the reactions comprising transfer of electrons commonly proceed via two or more sequential steps, reflecting the intrinsic complexity of enzymatic active sites, redox cofactors, and energy-conversion pathways^15–17^. Examples include multistep redox transformations in enzymes^13–15^, hormones and vitamins^16^, quinone-mediated electron and proton transports in living systems^9–11,16,18^, redox chains involved in crucial physiological processes such as respiration and photosynthesis^15,16^, and various coupled electron–proton transfer processes governing biological energy transduction^10,13–17^. In such complex systems, the electrochemical transformation does not take place as a single and “isolated” electron-transfer step, but instead it requires more complex models that account for chemical equilibria or regenerative (catalytic) reaction pathways.

One of the defining features of all bioelectrochemical mechanisms is their coupling with homogeneous chemical reactions, most commonly protonation-deprotonation equilibria^9–11,16–19^ conformational changes^16^, or ligand exchange reactions^18^. Occurrence of such chemical reactions inevitably leads to a dynamical redistribution of electroactive species near the electrode surface under voltammetric conditions. In addition, many biologically relevant redox systems exhibit catalytic behavior, where the product of an electron-transfer step is chemically converted back into an electroactive reactant, while giving rise to so-called electrochemical catalytic (regenerative) loops^16,18,20,21^. Such coupling between electron transfer, chemical equilibria, and regenerative chemical reactions is essential for sustaining redox processes in living systems^15^, but also helps in the designing of various electrochemical sensors and other electrochemical devices^16–18^.

Over the past few decades, a significant number of theoretical models describing different electrode mechanisms under various voltammetric techniques have been published^18,23^. These include the so-called simple “E” mechanism^18^, EC mechanism^18,21–23^, CE mechanism^21–23^, ECE mechanism^23^, consecutive two-step EE mechanism^7,18,21,23^, EEC mechanism^6^, one-step EC′ catalytic mechanism^16,18,20,22^, and the multistep catalytic EEC’ mechanism^2,5,8^, among others. These models provide valuable insights into various kinetic and thermodynamic aspects that are commonly met in important physiological and chemical systems. However, each theoretical framework is mainly restricted to a narrow mechanistic class, and it requires distinct mathematical treatment. To date, almost no single theoretical model has been developed under conditions of square-wave voltammetry, which unifies all common electrochemical mechanisms within one consistent mathematical formalism.

In this work, the ECrevEC′ reaction scheme is introduced and theoretically analyzed under conditions of square-wave voltammetry, serving as a comprehensive and unifying framework for the most common electrochemical mechanisms. The considered mechanism consists of two consecutive electron-transfer steps that are separated by a reversible homogeneous chemical reaction, and followed by an irreversible regenerative chemical step. By appropriate selection of thermodynamic and kinetic conditions, the ECrevEC′ mechanism can be reduced to most commonly encountered electrode mechanisms as limiting cases, including the simple E mechanism, EC and CE mechanisms, the ECE and EE sequences, as well as EC′ and EEC′ catalytic mechanisms. Importantly, this unification is achieved without altering the underlying reaction topology, allowing systematic exploration of all mentioned mechanistic transitions within a single theoretical model.

### Theoretical model

The electrochemical mechanism (ECrevEC’) considered in this work can be described by the sequences (a) to (d) included in overall reaction scheme (I):

1 $$\:{\mathrm{E}}_{1}:\:\:\:\mathrm{R}\mathrm{e}\mathrm{d}\:\:\rightleftarrows\:\:\mathrm{I}\mathrm{n}\mathrm{t}1\:+\:{\mathrm{e}}^{-}\:\:\:\left({k}_{\mathrm{s},1;}{\alpha\:}_{1}\right)$$

2 $$\:{\mathrm{C}}_{\mathrm{r}\mathrm{e}\mathrm{v}}:\:\:\mathrm{I}\mathrm{n}\mathrm{t}1\:\:\rightleftarrows\:\:\mathrm{I}\mathrm{n}\mathrm{t}2\:\:\:\left({{k}_{f},\:{k}_{b};\:K}_{\mathrm{e}\mathrm{q}}=\frac{{k}_{f}}{{k}_{b}};\:\:{k}_{\mathrm{c}\mathrm{h}\mathrm{e}\mathrm{m}}=\:{k}_{f}+\:{k}_{b}\right)$$

3 $$\:{\mathrm{E}}_{2}:\:\:\:\mathrm{I}\mathrm{n}\mathrm{t}2\:\:\rightleftarrows\:\mathrm{O}\mathrm{x}\:+\:{\mathrm{e}}^{-}\:\:({k}_{\mathrm{s},2},\:{\alpha\:}_{2})\:$$

4 $$\:{\mathrm{C}}^{{\prime\:}}\left(\mathrm{r}\mathrm{e}\mathrm{g}\mathrm{e}\mathrm{n}\mathrm{e}\mathrm{r}\mathrm{a}\mathrm{t}\mathrm{i}\mathrm{v}\mathrm{e}\right):\:\:\:\mathrm{O}\mathrm{x}\:+\:\mathrm{Y}\:\:\to\:\:\:\mathrm{I}\mathrm{n}\mathrm{t}2\:\:\:\:\:\left({k}_{\mathrm{c}}\right)$$

In the mathematical treatment of the electrochemical mechanism presented with reaction scheme (I), the concentration of each dissolved redox active species varies with time (*t*) and with the distance from the surface of working electrode (*x*) due to diffusion (defined by Fick’s second law), and due to occurrence of homogeneous chemical reactions in solution. In this section, we present a step-by-step formulation of the initial and boundary conditions under which the model is solved.

(i) At time *t* = 0, the bulk solution in electrochemical cell contains only the starting redox active form “Red”, whose concentration equals *c*(Red)_(x=0)_ = c*(Red), where c*(Red) is the bulk concentration of initial redox species “Red”.
(ii) At *t* = 0, and at a distance in the double layer next to the working electrode surface (this is assumed as *x* = 0 in the mathematical model), the concentrations of all other relevant redox active species equals zero, i.e.: *c*(Int1)_(x=0)_ = *c*(Int2)_(x=0)_ = *c*(Ox)_(x=0)_ = 0.
(iii) At *t* > 0, *x* →∝; *c*(Red)_(x_→∝) → c_*_(Red); *c*(Int1)_( x_→∝) = *c*(Int2)_( x_→∝) = *c*(Ox)_( x_→∝) → 0.
(iv) At *t* > 0, the electrode reactions E_1_ and E_2_ from reaction scheme (I) occur only at the working electrode surface (i.e. at *x* = 0) through the boundary conditions defined.

In such scenario, it holds that: *c*(Red)_(x=0)_ + *c*(Int1)_(x=0)_ + *c*(Int2)_(x=0)_ + *c*(Ox)_(x=0)_ = c^*^(Red);

Within this framework, the differential Eq. (5) to (8) given below define the contribution of the mass transport and that of the homogeneous chemical reactions going on in the diffusion layer.

### Differential equation relevant for initial “Red” species

5 $$\:\frac{{\partial \:c\left( {\mathrm{Re} d} \right)}}{{\partial \:t}} = D \cdot \frac{{\:\partial \:^{2} c\left( {\mathrm{Re} d} \right)}}{{\partial \:x^{2} }}$$

The differential Eq. (5) implies that “Red” form undergoes only mass transport by diffusion in solution, and there are no other terms related to occurrence of chemical reaction coupled to “Red” species. Its consumption/formation occurs exclusively via the first electron-transfer step (E_1_ in reaction scheme I) at the working electrode surface. Thus, away from the electrode, “Red” redox active species is transported solely by diffusion.

### Differential equation relevant to redox “Int1” species

6 $$\:\frac{{\partial \:c\left( {Int1} \right)}}{{\partial \:t}} = D \cdot \frac{{\:\partial \:^{2} c\left( {Int1} \right)}}{{\partial \:x^{2} }} - k_{f} \cdot c\left( {Int1} \right) + k_{b} \cdot c\left( {Int2} \right)$$

Redox species “Int1” is generated electrochemically from “Red” species at the working electrode via the first electrode transformation described as E_1_ in reaction scheme (I). Once being generated electrochemically, “Int1” species participates in reversible homogeneous chemical transformation of type Int1⇄Int2. The first term in differential Eq. (6) describes the diffusional mass transport of “Int1” redox species. The other two terms in Eq. (6) represent the net chemical consumption of “Int1” by the forward conversion to “Int2” (via the term − *k*_f_ *c*(Int1)), and its chemical regeneration from “Int2” by the backward catalytic chemical reaction that is incorporated in the term + *k*_b_ *c*(Int2). So, redox species “Int1” behaves as a diffusion-controlled intermediate redox form, whose concentration gets continuously redistributed via reversible chemical equilibrium.

### Differential equation relevant to redox species “Int2”

7 $$\:\frac{{\partial \:c\left( {{\mathrm{Int}}2} \right)}}{{\partial \:t}} = D \cdot \frac{{\:\partial \:^{2} c\left( {{\mathrm{Int}}2} \right)}}{{\partial \:x^{2} }} + {\mathrm{k}}_{{\mathrm{f}}} \cdot c\left( {{\mathrm{Int}}1} \right) - {\mathrm{k}}_{{\mathrm{b}}} \cdot c\left( {{\mathrm{Int}}2} \right) + {\mathrm{k}}_{{\mathrm{c}}} \cdot c\left( {{\mathrm{Ox}}} \right)$$

Redox form “Int2” is the key intermediate redox form that enters as initial reactant in the second electron-transfer step E_2_ of reaction scheme (I). Species “Int2” is formed chemically from “Int1” via the forward equilibrium reaction described with the term + *k*_f_ ·*c*(Int1), and depleted by the backward reaction to “Int1”, which is described by the term − *k*_b_·*c*(Int2) in Eq. (7). The additional term that appears in Eq. (7) (i.e., the term + *k*_c_·*c*(Ox)) represents the concentrational contribution obtained via the regenerative (catalytic) step Ox + Y → Int2. This is the so-called regenerative (catalytic) contribution that takes place under pseudo-first-order conditions. This is because it is assumed in the model that the regenerative substrate “Y” is present in large excess, and its concentration is included in the apparent rate constant “*k*_c_” in Eq. (7). The term + *k*_c_·*c*(Ox) in Eq. (7) implies that redox species “Ox” produced electrochemically, can be chemically converted back into redox species “Int2” in solution, thereby closing a regeneration loop that can enhance the overall flux in the second electron transfer step E_2_.

### Differential equation relevant to redox species “Ox”

8 $$\:\frac{{\partial \:c\left( {{\mathrm{Ox}}} \right)}}{{\partial \:t}} = D \cdot \frac{{\:\partial \:^{2} c\left( {{\mathrm{Ox}}} \right)}}{{\partial \:x^{2} }} - {\mathrm{k}}_{{\mathrm{c}}} \cdot c\left( {{\mathrm{Ox}}} \right)$$

Redox species “Ox” is formed electrochemically from “Int2” at the electrode surface via the second electron transfer step E_2_ in reaction scheme (I), after which it diffuses away from the surface of working electrode. In solution, however, species “Ox” is consumed by the irreversible chemical reaction with substrate “Y”, represented by the term: –*k*_c_ *c*(Ox) in Eq. (8). Thus, species “Ox” is a diffusion-transported final product that is continuously removed through homogeneous regeneration, while this consumption directly supplies the redox form “Int2” that is actually the initial reactant in E_2_.

Speaking in chemical context, the differential Eq. (5) to (8) describe the transformation of the initial redox species “Red” through two successive electron-transfer steps coupled by a reversible chemical equilibrium and followed by a regenerative reaction. In the definitions of Eq. (5) to (8), all concentration changes within the diffusion layer are accounted for under conditions of semi-infinite planar diffusion and homogeneous reaction kinetics.

By considering the semi-infinite planar diffusion concept, the faradaic current related to each electron-transfer step is proportional to the concentration gradient (diffusive flux) of the participating redox species at the working electrode surface. For the first electron transfer step E_1_ (Red⇄Int1 + e-), the current *I*1(*t*) is controlled by the interfacial fluxes of “Red” and “Int1” redox species. Analogously, for the second electron transfer step E_2_ (Int2 ⇄ Ox + e-), the magnitude of the current *I*2(*t*) is controlled by the interfacial fluxes of redox species “Int2” and “Ox”. In the simplest formulation with equal diffusion coefficients *D* (as considered in the current model), and identical number of electrons (*n* = 1) involved in both electron transfer steps, the boundary conditions at *x* = 0 can be written as:

For electron transfer step E_1_

9 $$\:x = 0:\:\:D \cdot \left( {\:\frac{{\partial \:c\left( {{\mathrm{Red}}} \right)}}{{\partial \:x}}} \right)_{{(x = 0)}} = \left( {\:\frac{{I1}}{{nFA}}} \right)$$

10 $$\:D \cdot \left( {\:\frac{{\partial \:c\left( {{\mathrm{Int}}1} \right)}}{{\partial \:x}}} \right)_{{(x = 0)}} = \left( {\:\frac{{ - I1}}{{nFA}}} \right)$$

For electron transfer step E_2_:

11 $$\:x = 0:\:\:D \cdot \left( {\:\frac{{\partial \:c\left( {{\mathrm{Int}}2} \right)}}{{\partial \:x}}} \right)_{{(x = 0)}} = \left( {\:\frac{{I2}}{{nFA}}} \right)$$

12 $$\:D \cdot \left( {\:\frac{{\partial \:c\left( {{\mathrm{Ox}}} \right)}}{{\partial \:x}}} \right)_{{(x = 0)}} = \left( {\:\frac{{ - I2}}{{nFA}}} \right)$$

In Eq. (9) to (12), *F* is symbol for the Faraday constant, while by *A* is assigned the active area of the working electrode, while *n* is number of electrons exchanged in both electron transfer steps (it is set to *n* = 1 for both electron transfer steps).

Equation (9) to (12) actually express the mass conservation at the working electrode-electrolyte interface. For each electron-transfer step, consumption of the electroactive reactant and formation of the electroactive product generate equal and opposite diffusive fluxes whose magnitudes are fixed by the corresponding faradaic currents.

For a complex two-step electrochemical mechanism such as the one considered in this work, it is frequently necessary to go beyond the reversible (Nernstian) approximation and to describe both electron-transfer steps using some kinetic concept. Under these conditions, the interfacial current densities are commonly defined by Butler-Volmer-type expressions^23^, which relate the currents *I*1 and *I*2 to the surface concentrations of the reacting species, and to exponential terms that are function of the applied potential difference. Within the framework of the proposed mechanism, the corresponding Butler-Volmer kinetic relations at the electrode surface (*x* = 0) can be formulated as follows:

13 $$\:I1/\left( {AF} \right)\: = \:{\mathrm{k}}_{{{\mathrm{s,1}}}} \cdot {\mathrm{exp}}(\alpha \:1 \cdot \phi 1) \cdot [c\left( {{\mathrm{Red}}} \right)_{{(x = 0)}} \: - \:{\mathrm{exp}}( - \phi 1) \cdot c\left( {{\mathrm{Int}}1} \right)_{{(x = 0)}} ]$$

14 $$\:I2/\left( {AF} \right)\: = \:{\mathrm{k}}_{{{\mathrm{s,2}}}} \cdot {\mathrm{exp}}(\alpha \:2 \cdot \phi \:2) \cdot [c\left( {{\mathrm{Int}}2} \right)_{{(x = 0)}} \: - \:{\mathrm{exp}}( - \phi 2) \cdot c\left( {{\mathrm{Ox}}} \right)_{{(x = 0)}} ]$$

In Eqs. (13) and (14), *Φ*1 and *Φ*2 are dimensionless potentials, defined as *Φ*1 = (*E*-*E*1)*F*/(*RT*), and *Φ*2 = (*E*-*E*2)*F*/(*RT*), where *E*1 and *E2* are formal redox potentials of Red/Int1 and Int2/Ox redox couples, respectively, while *R* is universal gas constant, and *T* is thermodynamic temperature. In addition, in Eqs. (13) and (14), with *α*1 and *α*2 are defined the electron transfer coefficients related to the first and the second electron transfer step, respectively (in the simulations, it is assumes that *α*1 = *α*2 = 0.5 for both electron transfer steps). Note that in the ECrev EC′ mechanism, the working electrode surface can act as a boundary where (formally) two distinct faradaic processes occur. The first current (*I*1) follows the interfacial conversion between “Red” and “Int1”, while the second current (*I*2) is due to the interfacial conversion between redox species “Int2” and “Ox”. The homogeneous reversible step Int1 ⇄ Int2, and the regenerative step Ox + Y→ Int2 take place in solution, and therefore they appear in the diffusion-reaction differential Eq. (5) to (8), whereas the electrode kinetics enter exclusively through the above boundary conditions (Eqs. 13 and 14). Using the Butler–Volmer kinetics in this mechanism is essential, since it is very rare to have fully reversible systems, so the Butler-Volmer formalism allows to simulate kinetic limitations of each electron-transfer step separately. The solution of differential equations under the conditions relevant to the ECrevEC’ mechanism has been performed following the protocols elaborated in^23^. Entire Mathcad working file that is designed to calculate square-wave voltammograms of ECrevEC’ mechanism is given in the Supplementary of this work. In^24^ one can find a detailed simulation protocols of electrode mechanisms in SWV in Mathcad program for free.

## Results and discussion

We consider in first part of this work a scenario where both electron transfer steps are separated for 300 mV or more at potential scale. Before starting the discussion of theoretical analyses, we first get insight into the dimensionless parameters affecting the voltammetric responses of analyzed ECrevEC’ mechanism, which are defined as follows:

- (a) Equilibrium constant of the intermediate chemical reaction *K*eq = *k*_f_/*k*_b_, where *k*_f_ and *k*_b_ are forward and backward rate constant of the chemical equilibrium Int1 ⇄ Int2;
- (b) Dimensionless rate parameter related to intermediate chemical reaction *K*_chem_ = (*k*_f_ + *k*_b_)/*f*, where *f* is symbol of SW frequency;
- (c) Dimensionless rate parameters related to the electron transfer at both steps, defined as: *K*1 = *k*_s.1_(*Df*)^−0.5^; *K*2 = *k*_s.2_(*Df*)^−0.5^, where *k*_s.1_ and *k*_s.2_ are the standard rate constants of electron transfers by the first and second electron transfer steps, respectively, while *D* is the diffusion coefficient (assumed to have value of 0.000005 cm^2^/s, equal for all redox species involved in electrode transformation);
- (d) Dimensionless rate parameter related to the regenerative (catalytic) chemical step Ox + Y → Int2, defined as: *K*_cat_ = (*k*_c_)/*f*, where *k*_c_ is the chemical regenerative rate parameter defined as: *k*_c_ = *k*_c_^’^ x *c*(Y), with *k*_c_^’^ symbolizing the real rate constant of the regenerative chemical reaction, while *c*(Y) is the concentration of regenerative substrate “Y” assumed to be in excess present in electrochemical cell. For the sake of simplicity in the theoretical model, interactions of species “Y” are restricted to species “Ox” only, resulting in regeneration of species “Int2”.

In addition, the simulated voltammetric patterns are function of the electron transfer coefficients related to both electron transfer steps *α*1 and *α*2 (set both to values of 0.5 in all simulations), temperature *T* (set to 298 K in all simulations), square-wave amplitude *E*sw (set to 50 mV in all simulations), number of electrons *n* = 1 equal for both electron transfer steps, and potential step d*E* (set to 10 mV in all simulations).

To get better visualization of the individual effects induced by particular kinetic and thermodynamic parameter, all simulated square-wave voltammograms are presented in three-dimensional format using the forward and backward current components.

### Voltammetric features of the ECrevEC’ mechanism at negligible rate of catalytic (regenerative) step

#### Effect of kinetic of intermediate chemical step expressed via *K*_chem_

**Fig. 1:**
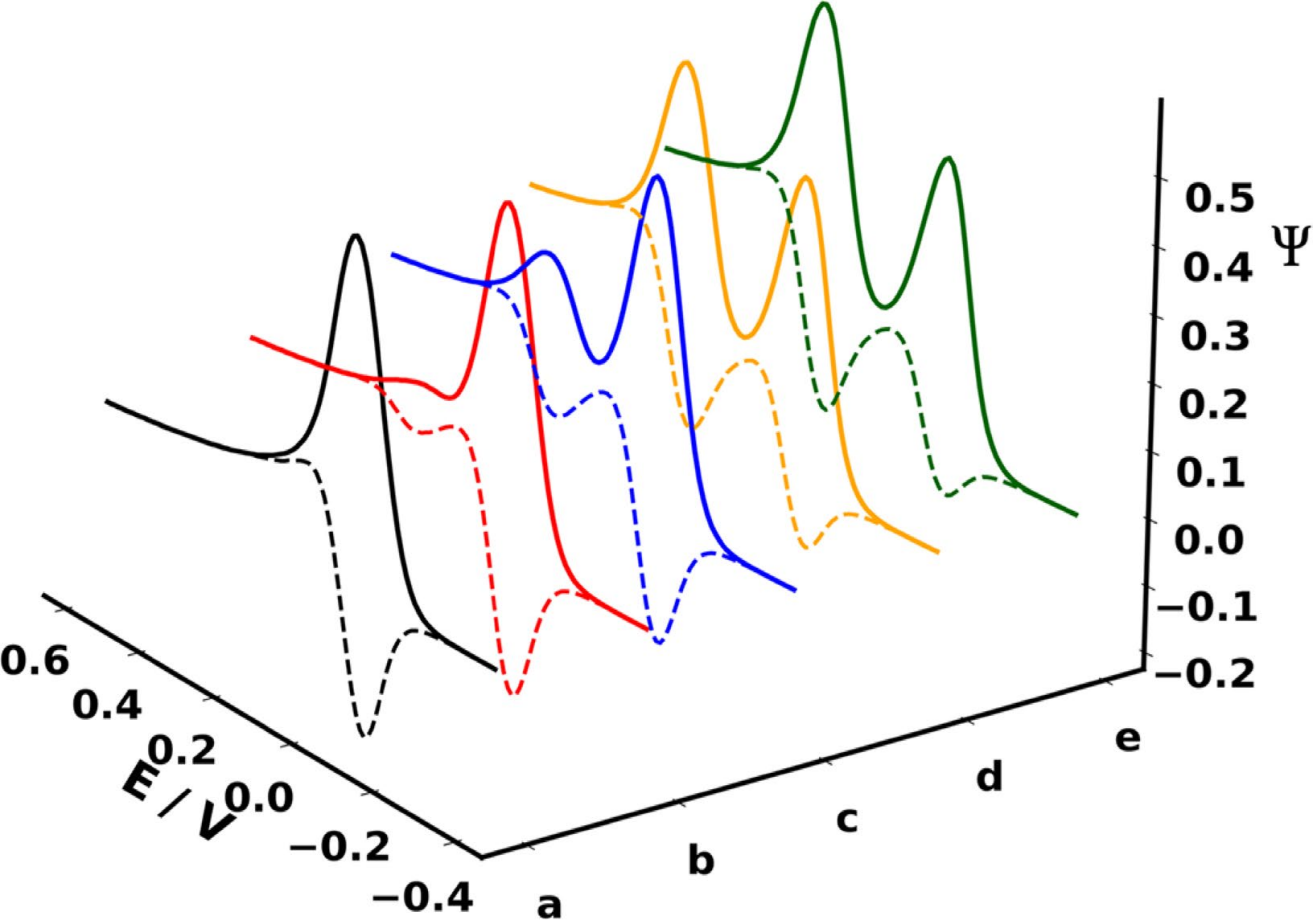
Three-dimensional square-wave voltammetric patterns of the ECrevEC′ mechanism simulated under negligible catalytic regeneration conditions (*K*_cat_ = 0.0001), illustrating the effect of the rate of the intermediate reversible chemical reaction (*K*_chem_) on the voltammetric responses. The equilibrium constant of the reversible chemical step was fixed at *K*eq = 0.5, while the chemical rate parameter was varied as *K*_chem_ = 0.0001 (a), 0.01 (b), 0.05 (c), 0.5 (d), and 1.0 (e). Other simulation conditions were: dimensionless kinetic rate parameters of both electron transfer step were identical and set to *K*1 = *K*2 = 0.316. In addition, identical values of both electron transfer parameters were used in all simulations, i.e., *α*1 = * α*2 = 0.5. Other conditions that were identical in all simulations were: number of exchanged electrons *n*1 = *n*2 = *n* = 1, temperature *T* = 298 K, square-wave amplitude *E*_sw_ = 50 mV, potential step d*E* = 10 mV, while the starting potential *E*s was set to − 0.3 V.

Under conditions where the catalytic regeneration associated with the product of the second electron-transfer step is effectively suppressed (roughly, for *K*_cat_ < 0.0005), and the equilibrium constant of the intermediate reversible chemical step is fixed at *K*eq = 0.5, the calculated voltammetric patterns reflect the intrinsic behavior of an ECrevE mechanism^3,23^. In this regime, the kinetic of the reversible homogeneous interconversion (quantified by the magnitude of *K*_chem_) primarily controls the features of the second electron-transfer step. At very small values of *K*_chem_ (voltammogram (a) in Fig. 1, calculated at *K*_chem_ = 0.0001), the homogeneous equilibrium is kinetically “frozen” on the voltammetric timescale. Consequently, the resultant voltammetric response is dominated by the first electron transfer, rendering the voltammogram’s features practically indistinguishable from a single-step “E” electrode mechanism^18,21,23,24^. When *K*_chem_ exceeds approximately 0.005 (curves (b) and (c) in Fig. 1), the reversible chemical step becomes sufficiently fast to dynamically couple the species generated in the first electron transfer step, while generating chemically the redox active species “Int2”. This sequence of events will lead to the formation and progressive development of a second peak positioned at more positive potentials. Simultaneously, the backward current associated with the first peak decreases concomitantly with the increase of *K*_chem_. This is a specific feature that reflects chemical redistribution and partial depletion of the product of first electron transfer during the time frame of the backward SW pulses^18,22,23^. Further increase of *K*_chem_ (curve (d) in Fig. 1, simulated for *K*_chem_ = 0.5) results in a well-developed two-peaks voltammetric pattern featuring strong electrochemical–chemical coupling. For *K*_chem_ > 0.5 (voltammogram (e) calculated for *K*_chem_ = 1), the voltammetric signatures approach the characteristics of an “EE mechanism”^7^, consistent with rapid establishment of the homogeneous equilibrium relative to the electrochemical timescale. Notably, increasing *K*_chem_ induces opposite potential shifts of the two processes. While the first peak is displaced toward more negative potentials, a hallmark of EC-type behavior^21–23^ arising from increased chemical perturbation of the first redox couple, in the same time the second peak shifts toward more positive potentials, typical of CE-type behavior^18,21–23^. Collectively, these trends that are nicely visible in the patterns of Fig. 1 demonstrate that, in scenario of negligible rate of catalytic step, systematic variation of *K*_chem_ alone drives a continuous mechanistic transition from an ECrevE-like response to an EE-like voltammetric pattern. The simultaneous but opposite peak shifts observed upon increasing of *K*_chem_ reveal the coexistence of EC- and CE-type effects embedded within the ECrevE electrochemical sequence in such scenario.

#### Effect of equilibrium constant of reversible chemical reaction

**Fig. 2:**
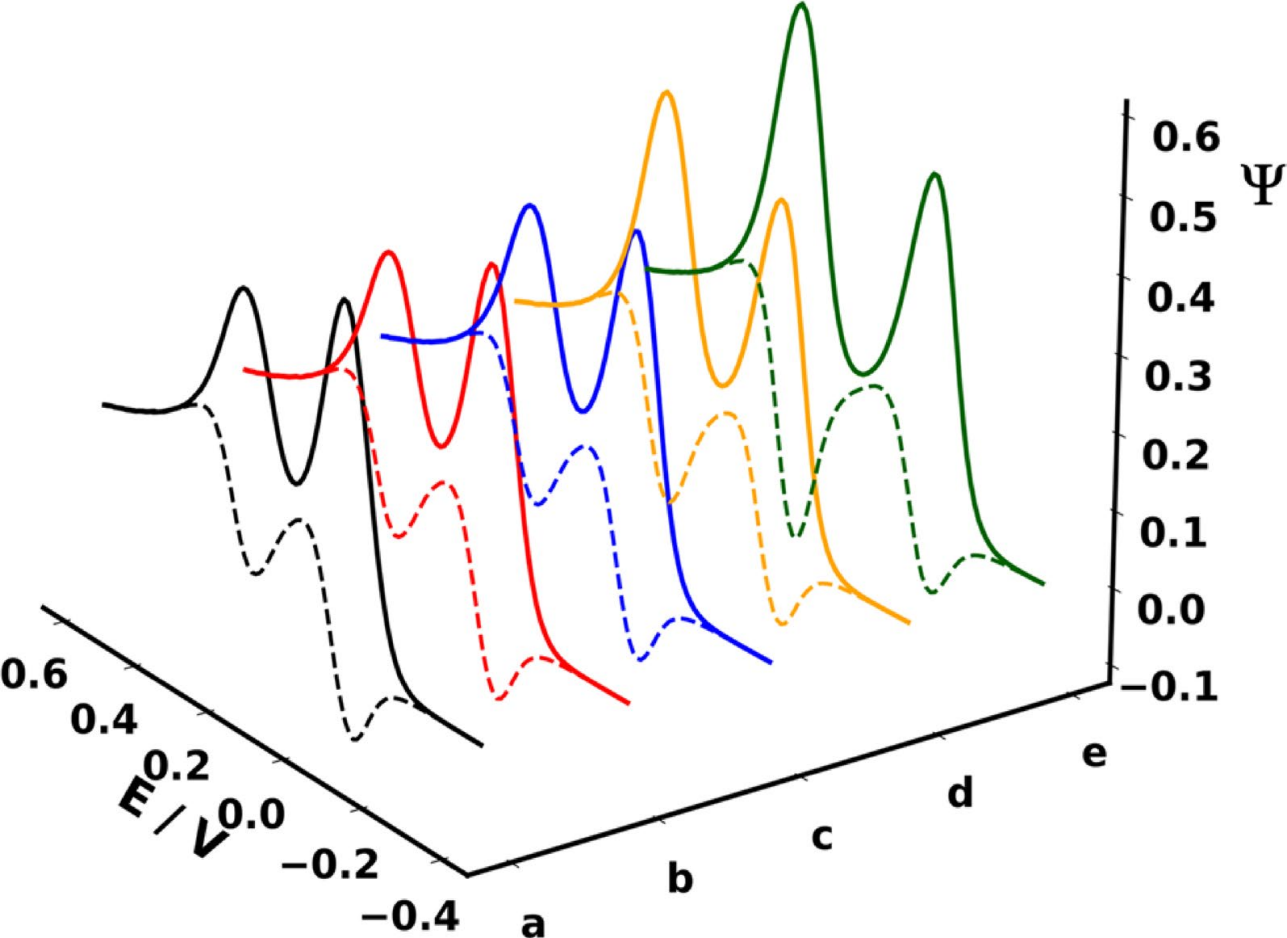
Three-dimensional square-wave voltammetric patterns of the ECrevEC′ mechanism simulated under negligible catalytic regeneration conditions (*K*_cat_ = 0.0001) and at a moderate value of the chemical rate parameter (*K*_chem_ = 0.5), illustrating the influence of the equilibrium constant of the intermediate reversible chemical reaction (*K*eq) the voltammetric responses. The equilibrium constant of the reversible chemical step was varied as *K*eq = 0.001 (a), 0.01 (b), 0.1 (c), 1 (d), and 10 (e), while other simulation conditions were identical to those used in Fig. 1.

Under conditions of negligible catalysis (*K*_cat_ < 0.0001) and at moderate-to-fast reversible chemical step (*K*_chem_ = 0.5), the ECrevEC′ scheme again reduces to an ECrevE mechanism, such that the voltammetric behavior is governed primarily by the thermodynamics of reversible homogeneous chemical step (*K*eq). It is important to note that changing the magnitude of *K*eq in ECrevE mechanistic scheme (at constant *K*_chem_, as it is in Fig. 2) enables analysis of voltammetric behavior of redox systems corresponding to distinct chemical equilibria operating under identical kinetic conditions. It should also be mentioned that *K*eq magnitude governs the efficiency of intermediate formation from the product of the first electron-transfer step, thereby controlling the supply to the redox active species “Int2” (i.e., the reactant in second electron-transfer step). So, for very small equilibrium constants (*K*eq = 0.001, curve (a) in Fig. 2), the chemical equilibrium strongly favors the initial form, and only a minor fraction of intermediate “Int2” is available for the second electron transfer. Consequently, the second peak of the voltammograms (at more positive potentials) is weak or it is poorly developed, while the voltammetric response is dominated by the first electron transfer step. As *K*eq increases through 0.01 (b) and 0.1 (c) in Fig. 2, the chemical equilibrium increasingly populates the intermediate species “Int2” and enhances the flux through the second electron-transfer step. As a consequence, a systematic growth of the second forward peak is observed, accompanied by a progressive redistribution of the backward currents. This behavior reflects stronger coupling between the chemical conversion following the product of first electron-transfer step and the generation of the “Int2” redox species that participates as reactant in the second electron-transfer step. In the thermodynamically balanced case (*K*eq ≈ 1, curve (d) in Fig. 2), the intermediate “Int2” is formed in substantial amount and the two-peaks voltammetric signature becomes clearly expressed. For *K*eq = 10 (curve (e) in Fig. 2), the equilibrium overwhelmingly favors the chemical formation of intermediate redox species “Int2”. In such scenario, one observes almost a maximal contribution of the chemical reaction in the second electron transfer. This will yield a voltammetric response that approaches the two-electron-transfer limit (i.e., the “EE mechanism”), which is expected for an effectively “intermediate-rich” ECrevE system. Concomitantly, variations in *K*eq influence the peak potentials of both processes. With increasing *K*eq, the first peak shifts toward more negative potentials because the equilibrium increasingly withdraws the product of the first electron-transfer step into the intermediate species “Int2”, resulting in an EC-like cathodic displacement of the first peak. In contrast, the second peak shifts toward more positive potentials as intermediate formation becomes favored, enabling the second electron-transfer step to proceed at more positive potentials^23^. Thus, at fixed *K*_chem_ and negligible rate of the regenerative step, systematic tuning of *K*eq modulates both the magnitude of the second peak, and also the relative positioning of the two peaks. This analysis provides a sensitive diagnostic tool for distinguishing whether reversible chemical step in considered mechanism is governed by thermodynamic control.

### Voltammetric features of ECrevEC’ mechanism under conditions of pronounced rate of regenerative chemical reaction

#### Scenario where *K*eq and *K*_chem_ of intermediate chemical reaction are both large: Effect of the magnitude of dimensionless catalytic rate parameter *K*_cat_

**Fig. 3:**
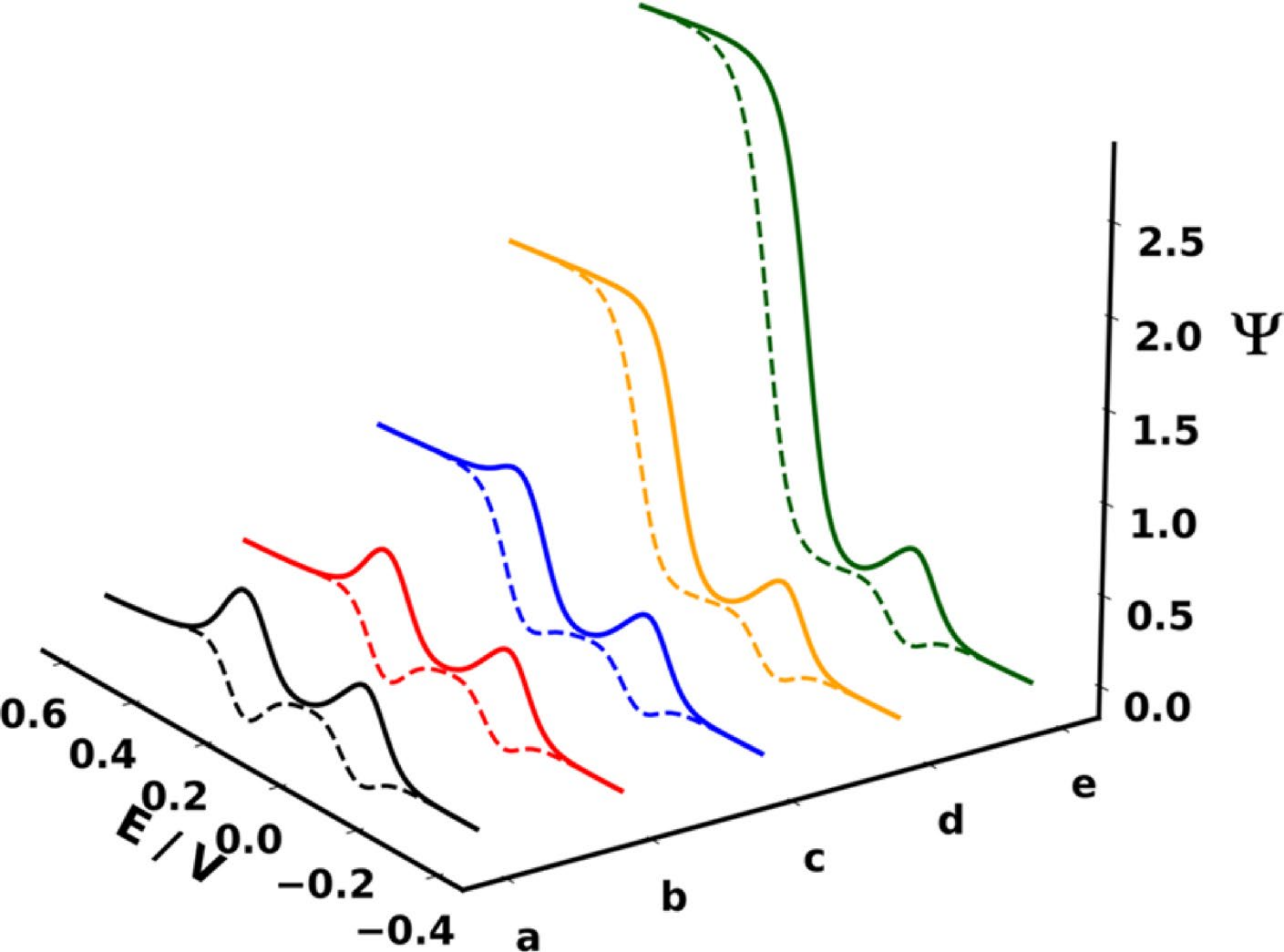
Three-dimensional square-wave voltammetric patterns of the ECrevEC′ mechanism simulated at large values of the chemical rate parameter (*K*_chem_ = 10) and the equilibrium constant (*K*eq = 5). Simulated curves illustrate the effect of the dimensionless catalytic rate parameter on the features of the voltammetric response. The catalytic rate parameter was varied as *K*_cat_ = 0.001 (a), 0.05 (b), 0.5 (c), 2.5 (d), and 7.5 (e). All other simulation conditions were identical to those used in Fig. 1.

At fixed and large magnitudes of equilibrium constant and that of the rate of chemical step (*K*eq = 5 and *K*_chem_ = 10, see Fig. 3), the reversible chemical interconversion between the product of first electron transfer step (“Int1”) and the redox active intermediate involved in second electron transfer step (“Int2”) is both thermodynamically biased toward the

“Int2” and kinetically fast on the voltammetric timescale. In such sequence of events, and in presence of pronounced rate of the catalytic reaction linked to the species involved in the second electron transfer step, the entire system effectively behaves as an EEC′ mechanism. In such scenario, the principal control parameter becomes the rate of the regenerative (catalytic) step, quantified via the magnitude of dimensionless catalytic rate parameter *K*_cat_. In this situation, two well-separated electron-transfer events are portrayed in two voltammetric peaks, while variation of *K*_cat_ from 0.001 (a) to 7.5 (e) and over, predominantly modulates the current response associated with the second electron-transfer process. At quite low *K*_cat_ values (Fig. 3a), catalytic turnover is slow and the second wave remains close to its non-catalytic magnitude. By progressively increasing *K*_cat_ (Fig. 3b–e), one sees effective recycling of the product of the second electron-transfer step (Ox) back into the electroactive intermediate (Int2), while producing the signature typical of EC′ catalytic mechanism^20–23^. In these circumstances, amplification of the forward current at the second peak, accompanied by suppression/attenuation of the corresponding backward (reverse) component occurs. As *K*_cat_ becomes progressively large (curves d–e in Fig. 3), the regeneration is sufficiently rapid that the second peak approaches a catalytic or “plateau-like” response^20^, reflecting near-continuous resupply of “Int2” species during the current measuring timeframe. In contrast, the first peak remains essentially unaffected across all region of applied *K*_cat_ series. This is because in the model considered, the catalytic loop is defined to be coupled to the chemistry following the second electron transfer and does not significantly perturb the interfacial balance of the first redox couple. So, under conditions where second peak is positioned ~ 300 mV positive in respect to the first peak, tuning the *K*_cat_ primarily controls the magnitude and reversibility of the second wave, while yielding voltammetric patterns characteristic of an EEC′ catalytic mechanism^9^.

#### Scenario in which *K*_chem_ of intermediate chemical reaction and *K*_cat_ of regenerative step have both moderate values: Effect of the magnitude of *K*eq

**Fig. 4:**
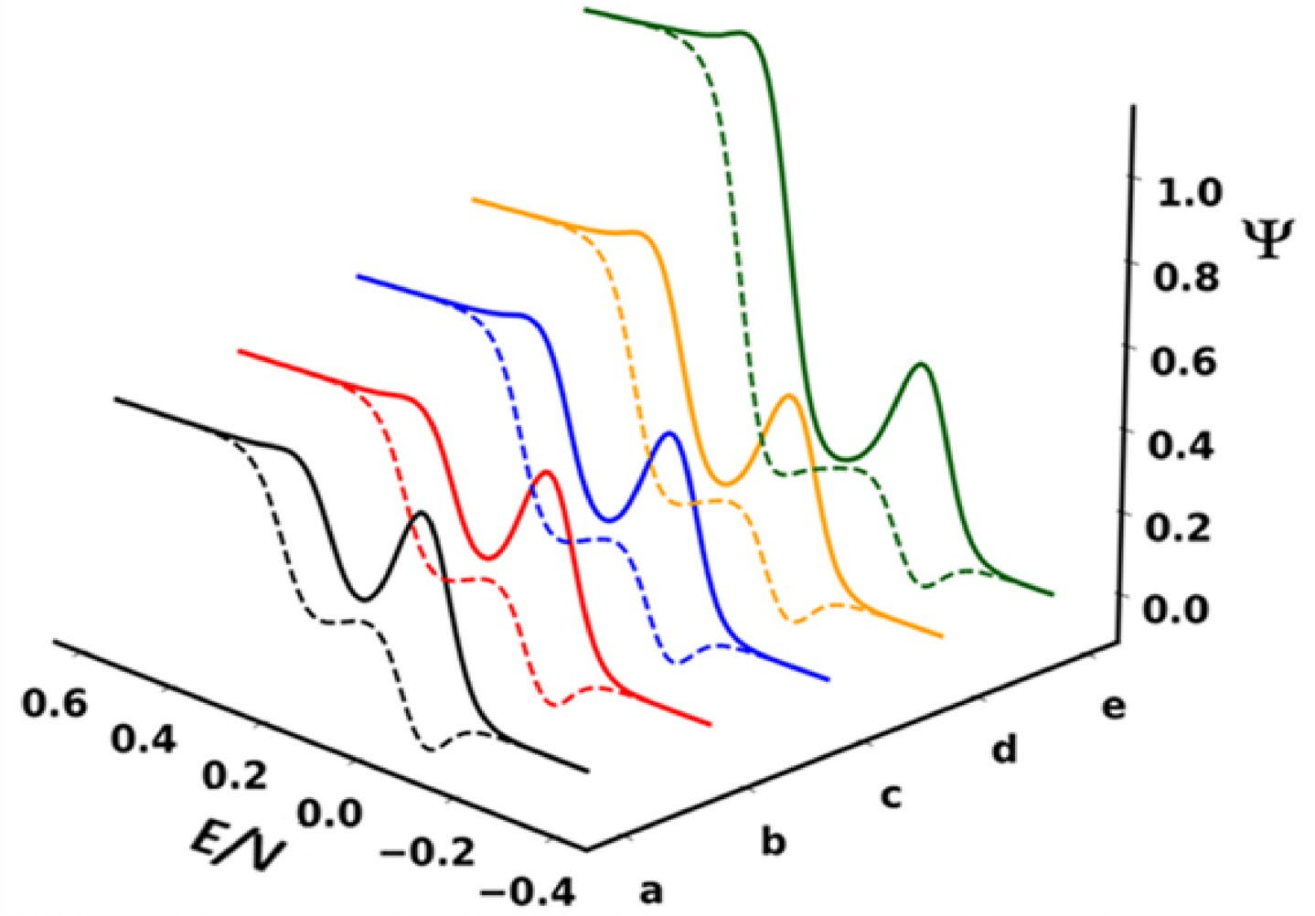
Three-dimensional square-wave voltammetric profiles of the ECrevEC′ mechanism illustrating the influence of the equilibrium constant (*K*eq) on the voltammetric responses, simulated under a moderate rate of the intermediate chemical step (*K*_chem_ = 0.5) and moderate kinetics of the regenerative reaction (*K*_cat_ = 1). The equilibrium constant was varied as *K*eq = 0.01 (a), 0.1 (b), 0.5 (c), 1 (d), and 10 (e). All other simulation conditions were identical to those used in Fig. 1.

The voltammetric patterns displayed in Fig. 4 feature a series of voltammograms of ECrevEC′ mechanism, simulated at identical kinetic conditions for the homogeneous step (*K*_chem_ = 0.5), and at identical kinetics of the regenerative reaction (*K*_cat_ fixed at a moderate value of 1). Under these conditions, only the equilibrium constant of the reversible intermediate chemical step is varied in the simulations, i.e., *K*eq was set to 0.01 (a), 0.1 (b), 0.5 (c), 1 (d), 10 (e) in Fig. 4. In that context, increasing magnitude of *K*eq progressively shifts the thermodynamic partitioning of the reversible chemical equilibrium toward the intermediate that feeds the second electron-transfer step. In this way, there will be an effective increasing of the availability of reactant for second electron transfer step (“Int2”). As a consequence, there will be enhancing extent to which the product of the second electron transfer step can be consumed through the catalytic regeneration loop. At very small *K*eq (Fig. 4a), formation of the intermediate is thermodynamically disfavored, so the second electron-transfer contribution is comparatively weak, and the regenerative loop is expressed only modestly. Accordingly, the second forward peak is small and the backward component remains relatively pronounced, indicating limited catalytic turnover. As *K*eq increases through (b) and (c) in Fig. 4, the intermediate species (Int2” becomes created in more significant extent, so the second peak becomes more clearly developed, and the catalytic signature of the second peak intensifies. In such scenario, the forward current associated with the second peak is amplified, while the corresponding backward current is progressively suppressed. This behavior is consistent with stronger catalytic recycling that sustains net flux through reactant for second electron transfer step and diminishes apparent reversibility on the return scan. In the regions of *K*eq ≈ 1 (Fig. 4d), intermediate formation is thermodynamically balanced and, combined with *K*_chem_ = 0.5, the coupled loop related to the second electron transfer step becomes strongly expressed, yielding a prominent second peak with clearly developed catalytic signature. At large *K*eq values (Fig. 4e), the equilibrium overwhelmingly favors the intermediate, maximizing the resupply to the reactant for second electron transfer step (“Int2”), while producing the most pronounced regenerative response. In such sequence of events, the second peak exhibits the significant forward current enhancement together with minimal reverse contribution, yielding a high catalytic turnover for the second electron transfer step. Thus, at fixed and moderate *K*_chem_ and *K*_cat_, variation of *K*eq acts as a purely thermodynamic control parameter that governs how efficiently the intermediate is generated chemically. Consequently, this will determine how strongly the catalytic regeneration of the product of the second electron transfer step is manifested in the voltammetric response.

### Scenario where *K*eq of intermediate chemical reaction and *K*_cat_ of regenerative step have both moderate values: Effect of the magnitude of *K*_chem_

**Fig. 5:**
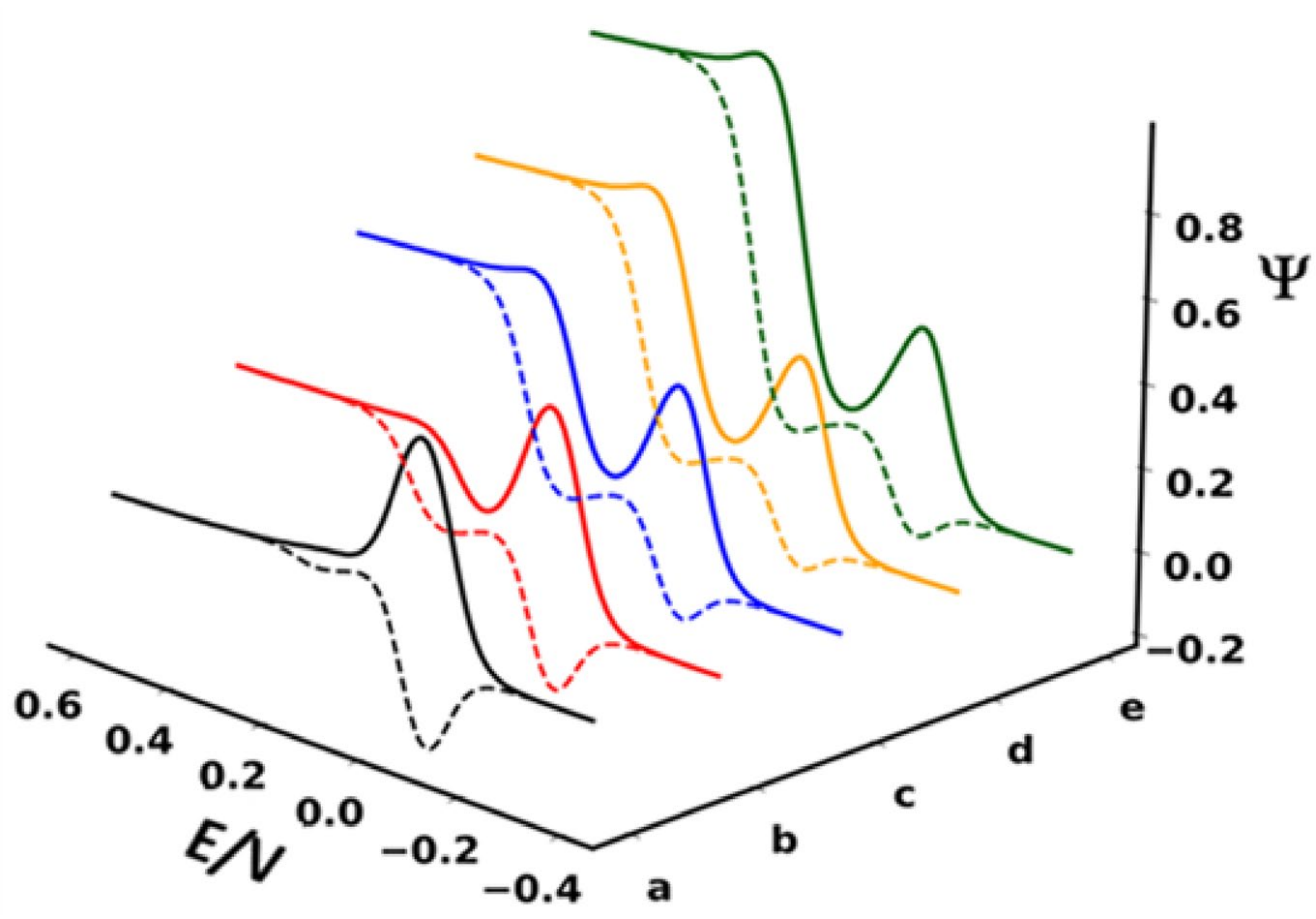
Three-dimensional square-wave voltammetric profiles of the ECrevEC′ mechanism illustrating the influence of the dimensionless chemical rate parameter (*K*_chem_ ) on the voltammetric responses, simulated under an equilibrium constant of the chemical step (*K*eq = 0.5) and moderate kinetics of the regenerative reaction (*K*_cat_ = 1). The magnitude of *K*_chem_ used in simulation was set to: *K*_chem_ = 0.01 (a); 0.1 (b); 0.5 (c); 1 (d); 10 (e). Other conditions use in simulation were identical as those reported in Fig. 1.

Shown in Fig. 5 is a series of SW voltammetric patterns that actually examine a series of ECrevEC′ mechanisms simulated at a moderate (fixed) equilibrium constant (*K*eq = 0.5) and moderate catalytic regeneration kinetics (*K*_cat_ = 1). In the analysis of voltammograms displayed in Fig. 5, the rate related to the reversible homogeneous intermediate chemical reaction has been systematically varied (*K*_chem_ **=** 0.01 (a), 0.1 (b), 0.5 (c), 1 (d), 10 (e)). At small *K*_chem_ values (Fig. 5a–b), the reversible chemical interconversion following the first electron-transfer step is kinetically sluggish, so the system converges to an ECrevE limit. In the course of these events, the first peak retains the quasi-reversible features, while formation of the intermediate feeding the second electron-transfer step is inefficient, resulting in a weakly expressed second peak, and only minor catalytic signature is observed. As magnitude of *K*_chem_ increases to intermediate values (curve c in Fig. 5), the rate of chemical step becomes competitive with that of diffusion and electron transfer, leading to a noticeable redistribution of concentration profiles near the working electrode surface. This situation will be portrayed in a progressive attenuation and slight cathodic shift of the first peak that reflects an EC-type behavior. Simultaneously, faster interconversion enhances the supply of reactant (redox species “Int2”) involved in the second electron-transfer step, thus allowing the catalytic regeneration loop to become more effective. At larger *K*_chem_ values (Fig. 5d-e), the homogeneous equilibrium is established rapidly on the voltammetric timescale, while producing strong coupling between the two electron-transfer steps. In such scenario, the backward current branch of first peak is further weakened and the peak gets kinetically distorted. In the same time, the second peak gets a pronounced catalytic signature, characterized by substantial forward-current amplification and marked suppression of the backward component that is typical of EC′ catalytic mechanism^20–23^. Thus, under conditions of fixed and moderate magnitudes of *K*eq and *K*_cat_, increasing *K*_chem_ plays a dual role: (a) it progressively loads the first electron-transfer step through EC-type chemical depletion, and (b) enabling at the same time efficient catalytic turnover at the second step by rapidly converting redox active material into the regenerative loop. Consequently, *K*_chem_ can be seen as a key kinetic parameter that controls both the perturbation of the first peak, and the expression of the catalytic efficiency of the second peak in the overall voltammetric response.

### Voltammetric features of ECrevEC’ mechanism when both electron transfers are separated for 100 mV or less at potential scale

**Fig. 6:**
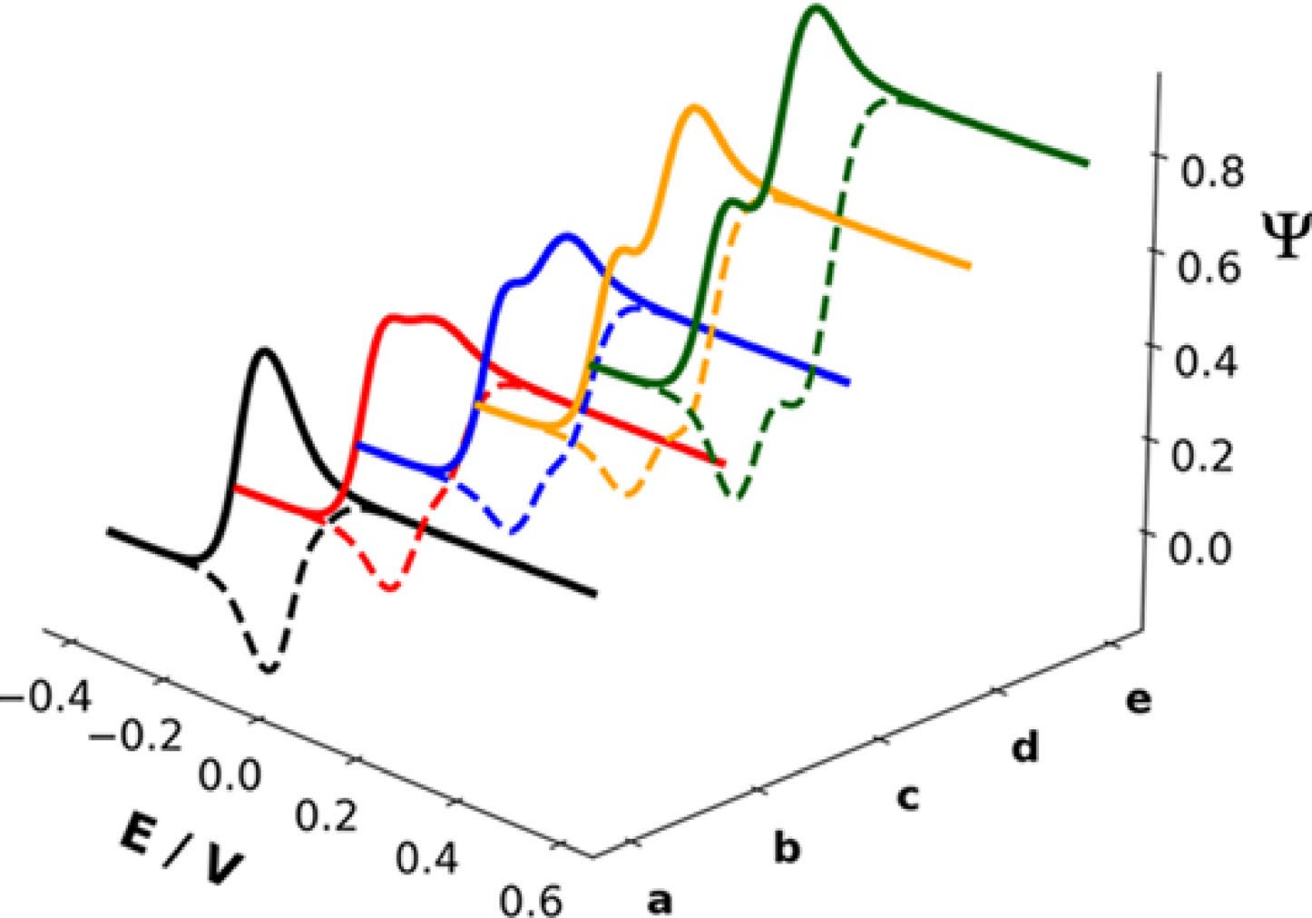
3-D SW voltammetric patterns showing the effect of the dimensionless chemical rate parameter *K*_chem_ on the resolution of both peaks, in scenario where the potential difference between both peaks was set to 100 mV. The magnitudes of *K*_chem_ applied in this series were: *K*_chem_ = 0.1 (a); 0.5 (b); 1 (c); 5 (d); 50 (e). For this series of simulations, magnitude of *K*eq was set to 0.5, while the magnitude of dimensionless catalytic parameter was se to *K*_cat_ = 1.0. All other simulation conditions were identical as those used in Fig. 1.

When the two electron-transfer steps of the ECrevEC′ mechanism are separated by approximately 150 mV or more on the potential scale (as demonstrated in Figs. 1 to 5), the corresponding square-wave voltammograms display two well-resolved peaks that can be correspondingly analyzed over a broad range of experimental conditions. In contrast, a significant analytical challenge arises when the potential separation between the two electron-transfer steps is reduced to about 100 mV or less. Figure 6 illustrates a series of square-wave voltammetric patterns for an ECrevEC′ system, simulated under conditions where the formal potential separation between the two electron-transfer steps is set to 100 mV. The simulations corresponding to Fig. 6 were performed at *K*eq = 0.5 and *K*_cat_ = 1, while the chemical rate parameter was varied in following range: *K*_chem_ = 0.1 (a), 0.5 (b), 1 (c), 5 (d), and 50 (e). At magnitudes of *K*_chem_ ≤ 0.1 (Fig. 6a), the rate of the reversible chemical step is not very pronounced on the timescale applied in SWV. The intermediate chemical redistribution is limited, so the two electron-transfer responses remain strongly overlapped. Therefore, at such a small potential separation, the two electron transfer steps typically merge and appear as a single composite peak, as seen in pattern (a) of Fig. 6. When *K*_chem_ is increased to 0.5 and 1 (Fig. 6b–c), the rate of chemical interconversion becomes competitive, so it perturbs the concentration balance near the working electrode surface. Upon increase of *K*_chem_, the first peak shifts towards more negative potentials, which is typical of an EC-type influence^21–23^. As a consequence, this shift reduces the overlap with the second process. Hence, the single composite peak begins to broaden and deform, while the second contribution related to the second electron transfer starts to emerge. At higher *K*_chem_ values (Fig. 6d–e), the chemical step is effectively fast, so the chemical equilibrium is established more rapidly during each SW cycle. In such scenario, the negative displacement of the first voltammetric peak becomes more pronounced, while the second process remains centered closer to its own formal potential. Consequently, the voltammograms progressively evolve into a clearly resolved two-peak pattern. This improves identification of both electron-transfer steps, even when their intrinsic separation is rather small. In this regime, the catalytic regeneration (*K*_cat_ = 1) further enhances the visibility of the second contribution, because the regenerated species sustains the flux through the second step. It is worth to mention in this context that although this scenario is theoretically achievable, it is difficult to implement it experimentally. In real ECrevEC’ systems with both electron transfer steps occurring at nearly identical potentials, the regenerative substrate “Y**”** is unlikely to affect only the second electron-transfer step. It will often interact with species involved in the first step as well. That coupling can blur the selective peak shifting assumed in the theoretical model and displayed in curves (c) to (e) in Fig. 6.

## Conclusions

The ECrevEC′ electrochemical mechanism represents a generalized two-electron transfer framework, in which two successive electrode reactions are coupled through a reversible homogeneous chemical step and a subsequent regenerative (catalytic) reaction. The voltammetric response of this mechanism is governed by a set of physically meaningful parameters, namely the equilibrium constant of the intermediate chemical step (*K*eq), its associated dimensionless chemical rate parameter (*K*_chem_), the kinetic rate constant of the catalytic regeneration (*K*_cat_), and the dimensionless rate parameters of the two electron-transfer steps (*K*1 and *K*2). Systematic variation of these parameters controls peak positions, peak separations, current magnitudes, peak shapes and the degree of reversibility or catalytic distortion observed in square-wave voltammograms. A major advantage of theoretical treatment of the ECrevEC′ mechanism is its unifying character. When the formal potentials of the two electron-transfer steps are separated by approximately 150 mV or more, the voltammetric response typically exhibits two well-resolved peaks. Under such conditions, the ECrevEC′ model provides a single theoretical platform from which the characteristic behavior of a wide range of classical mechanisms can be recovered as limiting cases, including the simple “E mechanism”, “EC” and “CE” mechanisms, the “ECE” sequence, the “EE” mechanism, the “EC′ catalytic mechanism” (holds for the second peak), and the “EEC′ catalytic mechanism” (see Table 1). This unification enables systematic comparison of different mechanistic regimes without changing the underlying reaction scheme, thereby offering a coherent framework for mechanistic classification and interpretation.

**Table 1:** Limiting situations and features of all possible mechanisms arising from ECrevEC′.

Region / limiting conditions	Conditions	Effective “observed” mechanism	Peak-wise interpretation/Remarks
A. Simple “E”(one-step electron transfer)	***K*eq < 0.001** and ***K***_**chem**_ **< 0.001** (independent of *K*_cat_)	**E** (simple one-electron)	The intermediate Crev step is both thermodynamically unfavorable and too slow, so the system behaves like a single electron transfer peak (no meaningful second-step chemistry expressed).
B. ECE (if there is slow kinetics of regeneration)	**0.01 ≤** ***K*eq ≤ 1**, ***K***_**chem**_ **> 0.01**, and *K*_cat_ **< 0.001**	**ECE** (overall)	In this window the reversible intermediate step is active (moderate *K*eq + sufficiently fast *K*_chem_), but regeneration is weak. In such scenario, the *first peak behaves like that of EC mechanism*, while the *second peak follows the patterns of CE mechanism*.
C. CrevEC′ at 2nd peak (regeneration step is “on”)	**0.01 ≤ Keq ≤ 1**, ***K***_**chem**_ **> 0.01**, and *K*_cat_ **> 0.005**	**CrevEC’ (second peak)**	Same base region as B, but now regeneration is strong enough: *the second peak converts from CE-like to CrevEC’* behavior (i.e., second electron transfer product is efficiently regenerated).
D. EE (highly expressed Crev step)	***K*eq > 10** and ***K***_**chem**_ **> 1**, with *K*_cat_ **≤ 0.001**	**EE**	The intermediate reversible chemistry is so favorable/fast that the system effectively behaves like *two sequential electron transfers (EE)*.
E. EEC′ (EE plus regeneration)	***K*eq > 10** and ***K***_**chem**_ **> 1**, with *K*_cat_ **> 0.01**	**EEC’**	Same as D, but regeneration is strong enough to impose *EC’ Character on the second peak*, so the net behaves as *EE mechanism with regeneration coupled to the second step*.

Significant values are in bold.

However, the situation becomes considerably more complex when the potential separation between the two electron-transfer steps is reduced to about 100 mV or less, because the corresponding voltammetric peaks tend to overlap and merge into a single composite response. Nevertheless, the present analysis demonstrates that even in this challenging regime, theoretical resolution of the two electron-transfer processes is possible under certain conditions, for example through appropriate tuning of the chemical rate parameter *K*_chem_, or by exploiting characteristic peak shifts and distortions induced by the coupled chemical and catalytic steps.

Anyway, the theoretical analysis of ECrevEC′ mechanism under voltammetric conditions offers significant conceptual and practical benefits. It provides a simplified yet physically rigorous framework for interpreting complex voltammetric responses arising from coupled electron-transfer and chemical processes, particularly in various bioelectrochemical systems. Using a single theoretical framework to describe diverse mechanisms enables direct comparison of kinetic regimes, reduces mechanistic uncertainty in many aspects, and clarifies the way in which chemical equilibria and regeneration processes influence voltammetric signals. As such, the ECrevEC′ mechanism elaborated in this work represents an important step toward a unified theoretical description of multistep electrochemical processes relevant to bioelectrochemistry, energy conversion, and redox catalysis.

Finally, it must be emphasized that in square-wave voltammetry the frequency acts as a global time-scale parameter that simultaneously affects all kinetic quantities involved in the ECrevEC′ mechanism, including *K*_chem_, *K*_cat_, and the electron-transfer rate parameters *K*1 and *K*2. For this reason, straightforward frequency-based kinetic analysis is generally not recommended for such complex electrode mechanisms^25^, as changes in frequency do not selectively probe individual steps. Instead, alternative experimental strategies are preferable.

## Supplementary Information

Below is the link to the electronic supplementary material.

Supplementary Material 1

## Data Availability

All data generated or analyzed during this study are included in this published article and its supplementary information file.
