# Supplementary material for: A generalized electrode mechanism unifying classical electrochemical pathways in square-wave voltammetry
Source: Sci Rep. 2026 Jul 7;16:21008. doi: 10.1038/s41598-026-60887-y (PMC13342351; doi:10.1038/s41598-026-60887-y)

**Supplementary Material**: MATHCAD working file related to the manuscript: A generalized electrode mechanism unifying classical electrochemical pathways in square-wave voltammetry

**Rubin Gulaboski, Ivan Bogeski**


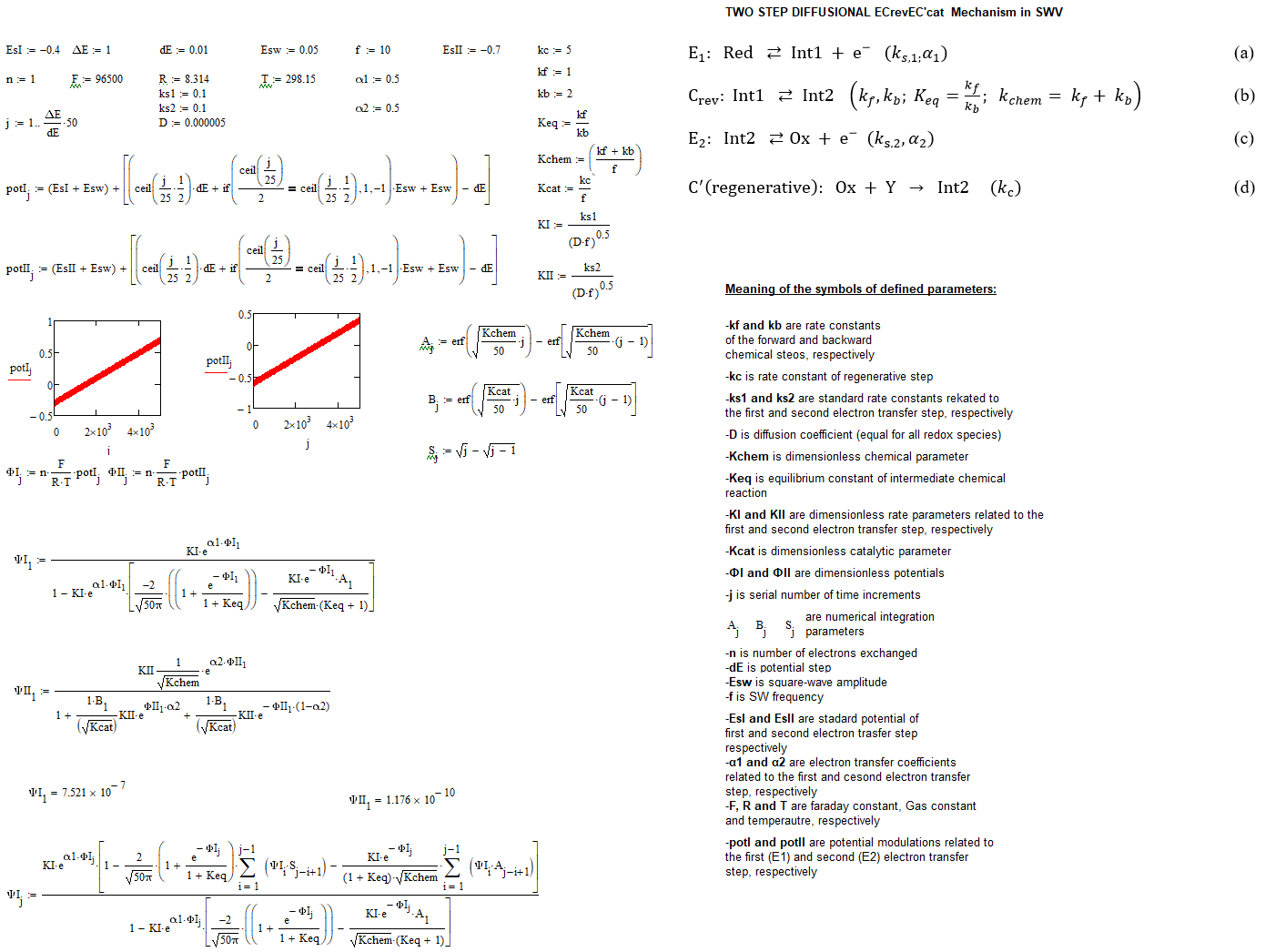


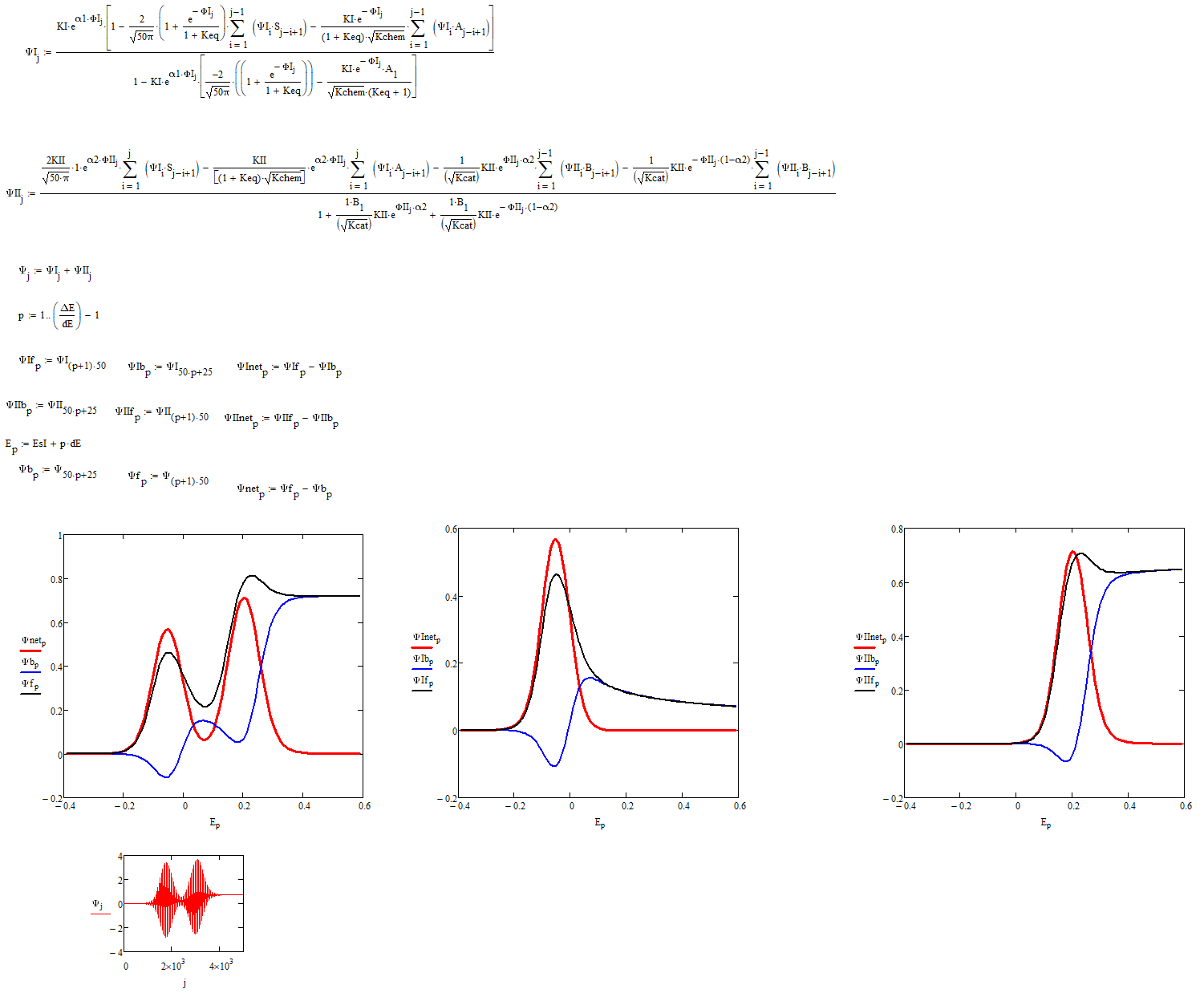

Supplement: Supplementary file 1 — Supplementary Material 1 [file 41598_2026_60887_MOESM1_ESM.docx]
